# PEDF Expression Is Inhibited by Insulin Treatment in Adipose Tissue via Suppressing 11β-HSD1

**DOI:** 10.1371/journal.pone.0084016

**Published:** 2013-12-18

**Authors:** Yinli Zhou, Fen Xu, Hongrong Deng, Yan Bi, Weiping Sun, Yi Zhao, Zonglan Chen, Jianping Weng

**Affiliations:** Department of Endocrinology and Metabolism, the Third Affiliated Hospital of Sun Yat-Sen University; Key Laboratory of Diabetology of Guangdong Province, Guangzhou, China; University of Texas Health Science Center at Houston, United States of America

## Abstract

Early intensive insulin therapy improves insulin sensitivity in type 2 diabetic patients; while the underlying mechanism remains largely unknown. Pigment epithelium-derived factor (PEDF), an anti-angiogenic factor, is believed to be involved in the pathogenesis of insulin resistance. Here, we hypothesize that PEDF might be down regulated by insulin and then lead to the improved insulin resistance in type 2 diabetic patients during insulin therapy. We addressed this issue by investigating insulin regulation of PEDF expression in diabetic conditions. The results showed that serum PEDF was reduced by 15% in newly diagnosed type 2 diabetic patients after insulin therapy. In adipose tissue of diabetic Sprague-Dawley rats, PEDF expression was associated with TNF-α elevation and it could be decreased both in serum and in adipose tissue by insulin treatment. In adipocytes, PEDF was induced by TNF-α through activation of NF-κB. The response was inhibited by knockdown and enhanced by over expression of NF-κB p65. However, PEDF expression was indirectly, not directly, induced by NF-κB which promoted 11β-hydroxysteroid dehydrogenase 1 (11β-HSD1) expression in adipocytes. 11β-HSD1 is likely to stimulate PEDF expression through production of active form of glucocorticoids as dexamethasone induced PEDF expression in adipose tissue. Insulin inhibited PEDF by down-regulating 11β-HSD1 expression. The results suggest that PEDF activity is induced by inflammation and decreased by insulin through targeting 11β-HSD1/glucocorticoid pathway in adipose tissue of diabetic patients.

## Introduction

Pigment epithelium-derived factor (PEDF) is a 50 kDa glycoprotein that was originally identified in 1991 as a growth factor secreted by retinal pigment cells [1,2]. PEDF is expressed in many tissues, including adipose, brain, spinal cord, eye, plasma, bone, prostate, pancreas, heart, liver and lung [3]. It has been reported to have many functions in regulating proliferation and differentiation of endothelial cells, in which PEDF inhibits angiogenesis and involves in inflammatory response [4–7]. PEDF regulates glucose metabolism in obesity in animal studies [8,9]. However, the significance of modulating PEDF expression remains unknown in the treatment of type 2 diabetes in clinical setting. 

In obese patients, adipose tissue is a major source of PEDF [9]. PEDF is the most abundant protein found in the culture medium of adipocytes (3T3-L1) [10]. Recently, several reports have indicated that the expression of PEDF is negatively associated with insulin sensitivity [11–13]. In mice, the infusion of PEDF leads to insulin resistance (IR) by inducing adipose tissue lipolysis [10]. In humans, an increase in serum PEDF is associated with the development of insulin resistance, and a reduction in serum PEDF is associated with improved insulin sensitivity following weight loss [11–14]. PEDF regulates adipocyte differentiation and lipolysis. In cell culture, PEDF inhibits the differentiation of pre-adipocyte 3T3-L1 cells by activating the (mitogen-activated protein kinases) MAPK/ (extracellular-signal-regulated kinases) ERK signaling pathway [15] and induces lipolysis in differentiated adipocytes in an adipose triglyceride lipase (ATGL) dependent manner [9]. The suppression of adipocyte activities by PEDF may contribute to the ectopic lipid deposition and insulin resistance in obesity. PEDF is positively associated with tumor necrosis factor-α (TNF-α) in serum of type 2 diabetic patients in Japanese [12]. Others found that PEDF expression is induced by dexamethasone (Dex) in the human trabecular meshwork [16]. However, the exact mechanism by which PEDF expression is increased in the adipose tissue remains unknown in obesity.

Previously, we reported that intensive insulin therapy improved insulin sensitivity in newly diagnosed type 2 diabetic patients [17]. Compared to treatments using oral hypoglycemic agents, insulin therapy enhanced the recovery of β-cell function and prolonged glycemic remission in the patients [17]. The therapy prevented glucotoxicity and lipotoxicity in patients with type 2 diabetes [17,18]. However, it is unknown if PEDF is involved in the improved insulin sensitivity after insulin therapy. Thus, we examined PEDF response to insulin both in type 2 diabetic patients and diabetic models. Since evidence showed PEDF was positive related with insulin resistance, we hypothesize that insulin treatment may down-regulate PEDF expression and then lead to the improved insulin sensitivity. It’s the first time to investigate whether insulin treatment could affect PEDF expression in serum and adipose tissue and to explore the mechanisms through which insulin action regulates PEDF. 

To address this hypothesis, we conducted studies in human with type 2 diabetes, animal models and adipocytes. In which we found that the serum PEDF was reduced by the insulin therapy in type 2 diabetic patients. In the mechanism, insulin suppressed PEDF expression in adipocytes probably by inhibiting 11β-HSD1 expression. We propose that inhibition of PEDF and 11β-HSD1 expression may involve in the mechanism of insulin sensitization by the insulin therapy. 

## Methods

### Cell culture and treatments

3T3-L1 cells (American Type Culture Collection, Manassas, VA) were cultured in dulbecco’s modified Eagle’s medium (DMEM) supplemented with 4 mM L-glutamine, 100 U/mL penicillin, 100 g/mL streptomycin, and 10% fetal bovine serum (FBS) at 37 °C in a humidified atmosphere containing 5% CO_2_. Two days after the cells reached confluency, the medium was replaced with an adipogenic cocktail containing 10% fetal bovine serum, 10 μg/mL insulin, 4 μg/ml Dex (Sigma, St. Louis, MO), and 0.5 mM 3-Isobutyl-1-methylxanthine (IBMX) (Sigma, St. Louis, MO). After three days, the cells were further differentiated in a culture medium containing 10% FBS and 10 μg/ml insulin for 4 days; the medium was changed every 2 days. Adipocyte maturation was confirmed by oil-red O staining of lipid droplets in the cells. To induce PEDF expression, the cells were treated with 100 nM Dex for 8 days or 20 nM TNF-α for 4 days. The effects of insulin on PEDF expression were detected by treatment of 100 nM insulin alone or simultaneously together with 100 nM Dex, or 20 nM TNF-α for 4 days, or 8 days. The effects of TNF-α or Dex on PEDF mRNA expression were measured by treatment for 30 min, 1, 2, 3, 6 hours. All the in vitro experiments in the study were repeated 3 times. 

### Animal models

The animal studies were approved by the Animal Care and Use Committee (IACUC) at Sun Yat-Sen University and were conducted in accordance with the principles of laboratory animal care (National Institutes of Health publication no. 85-23, revised 1985). Male Sprague-Dawley(SD) rats (7-8 weeks, approximately 200 g) were purchased from Southern Medical University (Guangzhou, China), and type 2 diabetes mellitus was induced as reported previously [18]. The control rats were fed a low-fat diet (64% carbohydrate, 26% protein, and 10% fat), and the diabetic rats were fed a high-fat diet (HFD) (32% carbohydrate, 14% protein, and 56% fat) for 5 weeks. In the diabetic group, the mice were fasted overnight, and hyperglycemia was induced by one i.p. injection of 40 mg STZ (Sigma, St Louis. MO) in 0.1 M citrate-buffered saline (pH 4.2) per kg body weight. The control group was treated with the citrate-buffered saline. Insulin treatment was administered in the diabetic rats as described previously [18]. On the third day after the STZ injection, weight- and glucose-matched diabetic rats were divided randomly into 4 groups with 6 animals per group as follows: (a) NC group: normal control; (b) DM group: untreated diabetic rats; (c) EI group: early intensive insulin treatment group treated with neutral protamine hagedorn (NPH) insulin for 3 weeks after the STZ injection; and (d) EG group: diabetic rats treated with gliclazide (a drug which improve insulin secretion) for 3 weeks after the STZ injection. The gliclazide group was served as a negative control for comparison with insulin treatment.

Forty 10-week old male C57BL/6J mice were purchased from the animal center of Guangdong Province. The mice were injected (i.p.) with TNF-α (6 μg/kg/d) or Dex (0.2 mg/kg/d) to induce PEDF expression. After TNF-α or Dex injection, the total RNA from adipose tissue were extracted and the serum were collected at the time point of 2, 6, 24, 48, 96, 192 hours. The male adipocyte fatty acid binding protein 2 (aP2)-p65 mice were reported elsewhere [19]. The mice had free access to water and food. The mice were ear-punched for identification and housed 3-4 mice/cage.

### PEDF in normal controls and T2D patients before and after insulin treatment

22 newly diagnosed type 2 diabetes patients (10 men and 12 women) and 29 healthy controls (14 men and 15 women) were enrolled into the study. The exclusion criteria included having severe acute or chronic complications, receiving anti-diabetic treatment before the study, having other severe diseases, or taking anti-hyperlipidemic agents. The healthy controls were 45.8±9.0 years and the type 2 diabetic patients were 51.4±8.0 years old. The time since diagnosis of diabetes was 1-30 days, with the starting level of HbA1c 11.5±2.1%. The last dose of insulin glargine was delivered ≥34 hours before the blood sample used to measure PEDF was collected. The blood was collected under similar conditions: following overnight fast, between 8:00-9:00 am at next morning. All participants provided their written informed consent to participate in this study. Serum samples were obtained before and after 14 days of glargine therapy. Subjects underwent intravenous glucose tolerance tests (IVGTTs) using 25g of glucose (50 ml of 50% glucose solution) before and after glargine therapy. Serum samples were obtained before (0 min) and 1, 2, 4, 6, and 10 min after injection for insulin and C-peptide determination. The levels of PEDF and adiponectin in the serum were determined using enzyme-linked immunosorbent assay (ELISA) kit (Chemicon CYT420, CYT282, Billerica, MA). 

### Real time RT-PCR

Total RNA was used to generate cDNA at 2μg/sample. Reverse transcription was performed using the cDNA Synthesis Kit and SYBR® Green PCR Master (TakaRa, Japan), as previously described [18]. The primer sequences used in this study were shown in Table 1.

**Table 1:** Primer sequences used in this study.

Name	GenBank #	Sequence	Product size (bp)
IL-6F	NM_012589	CCGGAGAGGAGACTTCACAG	161
IL-6R		ACAGTGCATCATCGCTGTTC	
TNFAF	NM_012675	ACTCCCAGAAAAGCAAGCAA	211
TNFAR		CGAGCAGGAATGAGAAGAGG	
PEDFF	NM_177927.2	GTGGGCAACCAAGTTTGACT	156
PEDFR		AGGGGCAGGAAGAAGATGAT	
GAPDHF	NM_017008.3	ACCACAGTCCATGCCATCAC	144
GAPDHR		TCCACCACCCTGTGCTGTA	
11βHSD1F	NM_005525.2	TTGCTTTGGATGGGTTCTTC	162
11βHSD1R		AGCTCCCCCTTTGATGATCT	
hGAPDHF	NM_002046.3	GAGTCAACGGATTTGGTCGT	189
hGAPDHR		TTGATTTTGGAGGGATCTCG	

### Western blotting

Protein concentration was determined using the Bio-Rad Protein Assay kit (Bio-Rad, Hercules, CA). For the Western blots, proteins (40μg/sample) were separated by SDS-PAGE and blotted with specific antibodies. The antibodies were anti-PEDF (#7280, Millipore, USA), anti-NF-кB P65 (#3034, Cell Signaling Technology, Danvers, MA), and anti-phospho-NF-кB P65 (#3037, Cell Signaling Technology, Danvers, MA). A GAPDH antibody (#4967, Cell Signaling Technology) was used as the protein loading control. 

### Transfection and reporter assay

In this study, we used two PEDF luciferase reporters that differ in the promoter length. The short promoter hPEDFP1 (−1721/+38) and the long promoter hPEDFP2 (−4687/+38) were kind gifts of Dr. Kenichi Nakahama (Tokyo Medical and Dental University). 

In the transfection, fully differentiated 3T3-L1 cells were co-transfected with 0.5 μg of the PEDF reporter plasmid and 2 ng of the internal control plasmid pRL-CMV using Fugene HD (Roche, Indianapolis, IN). In Dex treatment, the cells were treated with Dex (100 nM) for 16 h to induce the promoter activity before being harvested for the dual-luciferase assay. 

To test the role of NF-κB in regulating PEDF expression, NF-кB p65 was knocked down in 3T3-L1 adipocytes using miRNA, which was delivered by the lentivirus CN45. The lentivirus was prepared by GeneChem (Quebec, Canada). NF-κB p65 was over expressed using a p65 expression vector as described elsewhere [20]. 

### Ethics Statement

This work was approved by the ethical committees of the Third Afﬁliated Hospital of Sun Yat-sen University, and all subjects were given informed consent. All participants provided their written informed consent to participate in this study.

### Statistical analysis

The results are expressed as the mean ± SEM. The unpaired Student’s t-test was used to determine statistical significance, which was set to P ≤0.05. ANOVA analysis was used to compare the gene expression in subjects of different groups. Calculations were performed using the statistical program SPSS version 13.0 (Statistical Package for the Social Sciences, SPSS, Inc., Chicago, IL).

## Results

### PEDF was reduced by insulin treatment in T2D patients

**Figure 1:**
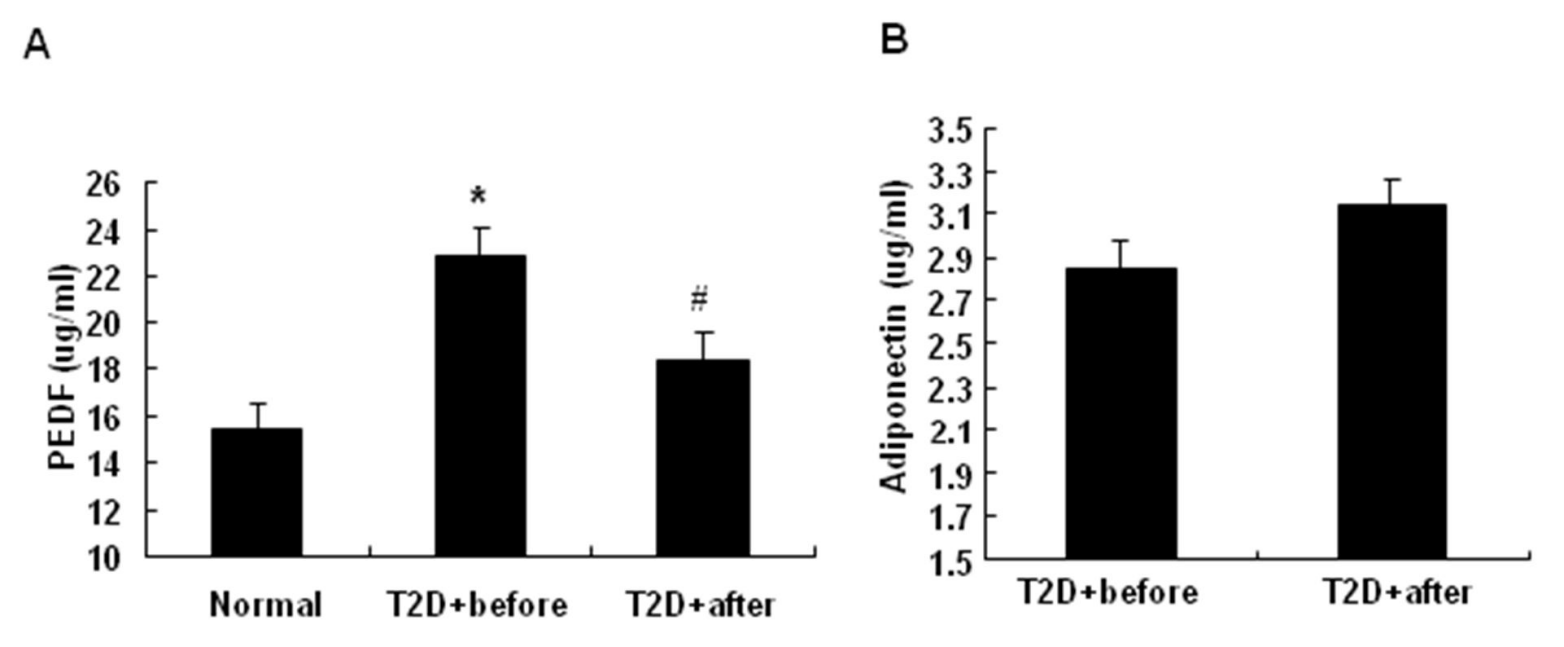
PEDF levels were reduced by insulin treatment in serum of T2D patients. The serum samples were obtained from 29 healthy controls (14 men and 15 women) and 22 newly diagnosed type 2 diabetic patients (10 men and 12 women) before and after 14 days of insulin glargine therapy. Serum PEDF and adiponectin were determined by ELISA. *A*: The effects of intensive insulin therapy on the serum PEDF level in normal controls and newly diagnosed T2D patients before and after insulin therapy. *B*: The effects of intensive insulin therapy on the serum adiponectin level in newly diagnosed T2D patients before and after insulin therapy. The data are presented as the mean ± SEM of four independent experiments. “*” indicates P<0.05 compared with normal control. “#” indicates P<0.05 compared with T2D patients before insulin therapy. “T2D+before” means T2D patients before insulin therapy, “T2D+after” means T2D patients after insulin therapy.

Our previous study indicates that intensive insulin therapy improves insulin sensitivity [17]. To test the role of PEDF, we measured the serum PEDF levels in 29 healthy persons and 22 newly diagnosed type 2 diabetic (T2D) patients. Serum PEDF was significantly higher in the patient than that in healthy subjects (Figure 1A). PEDF levels were compared in those patients before and after 14 days of insulin therapy. The PEDF levels were significantly reduced in the patients by the therapy (Figure 1A). In the study, serum adiponectin was monitored. The insulin therapy modestly induced adiponectin, but the change is not significant (Figure 1B). Other information of the patients before and after the insulin therapy is shown in Table 2. HOMA-B in T2D patients increased from 25.8±17.3 to 170.0±118.9 after insulin therapy, which indicates the β cell function of T2D patients recovered after insulin treatment. The results suggest that insulin therapy decreases PEDF in the blood of type 2 diabetes patients. 

**Table 2:** Clinical information of the non-diabetic controls and T2D patients before and after insulin treatment.

	Non diabetic controls	T2D before insulin treatment	T2D after insulin treatment
BMI	20.4±1.7	24.9±2.5	24.8±2.5
Glucose (mmol/L)	4.8±0.4	12.9±2.8	5.9±1.4*
2h glucose (mmol/L)	5.7±0.9	5.7±0.9	9.8±2.2*
TC (mmol/L)	5.0±0.8	5.9±1.0	5.3±1.0*
TG (mmol/L)	1.0±0.6	3.1±1.8	1.7±0.8*
HOMA-B	143.0±59.3	25.8±17.3	170.0±118.9*

TC: Total cholesterol, TG: Triglycerides, BMI: Body Mass Index, HOMA-B: HOMA-Beta. HOMA-B=20 × INS / (GLU-3.5), the blood tested for insulin and glucose was collected following overnight fast, between 8:00-9:00 am. “*” indicates P < 0.05 compared with the group before insulin treatment.

### PEDF expression was inhibited by insulin in diabetic rats

**Figure 2:**
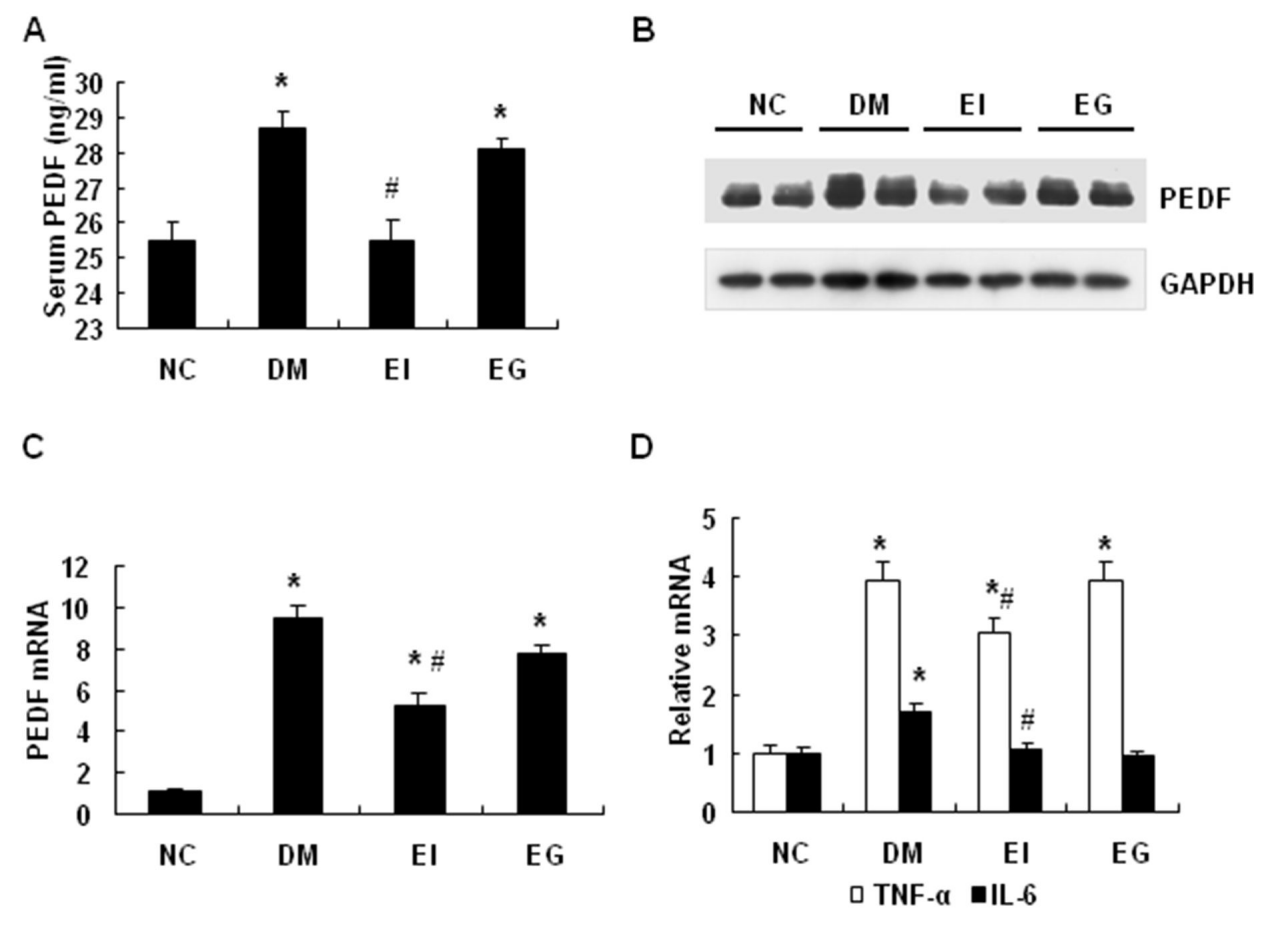
PEDF expression was inhibited by insulin in diabetic rats. *A*: Insulin treatment decreased the serum PEDF in type 2 diabetic SD rats. *B*: Insulin treatment decreased PEDF protein in the adipose tissue of type 2 diabetic SD rats. *C*: Insulin treatment decreased PEDF mRNAs in the adipose tissue of type 2 diabetic SD rats. *D*: Insulin treatment decreased IL-6 and TNF-α mRNA expression in the adipose tissue of SD rats. White bars = TNF-α; black bars = IL-6. The data are presented as the mean ± SEM of four independent experiments. “*” indicates P<0.05 compared with the control group. “#” indicates P<0.05 compared with diabetic group. *NC*, normal control; *DM*, diabetic rats with no insulin therapy; *EI*, diabetic rats treated with insulin during early intervention study; EG, diabetic rats treated with gliclazide during early intervention study.

To investigate the insulin activity further, we examined PEDF in type 2 diabetic rats following the insulin therapy. The diabetic rats were made as described elsewhere [18] and divided into three groups, which included untreated diabetic (DM), insulin-treated (EI) and gliclazide-treated (EG) groups. Serum PEDF was elevated in the diabetic group relative to the nondiabetic group (NC) (Figure 2A). The elevation was reduced by insulin, but not by gliclazide in the diabetic rats (Figure 2A). The adipose tissue is a major source of serum PEDF [10]. In the adipose tissue, PEDF protein and mRNA were significantly increased in the untreated diabetic group, and the levels were decreased by the insulin treatment (Figure 2, B-C). Gliclazide, a sulfonylureas hypoglycemic drug that stimulates insulin secretion in β-cells, had no significant effect on PEDF in the adipose tissue of diabetic rats (Figure 2, B-C). The expression of inflammatory cytokines TNF-α and Interleukin-6 (IL-6) was examined in the adipose tissue. Both cytokines were increased by diabetes and reduced by insulin (Figure 2D). These data suggest that PEDF is increased both in the blood and in the adipose tissue of diabetic rats. The increase is decreased by insulin therapy, but not by gliclazide therapy. Insulin also inhibits expression of the inflammatory cytokines. 

### PEDF expression was inhibited by insulin in adipocytes

**Figure 3:**
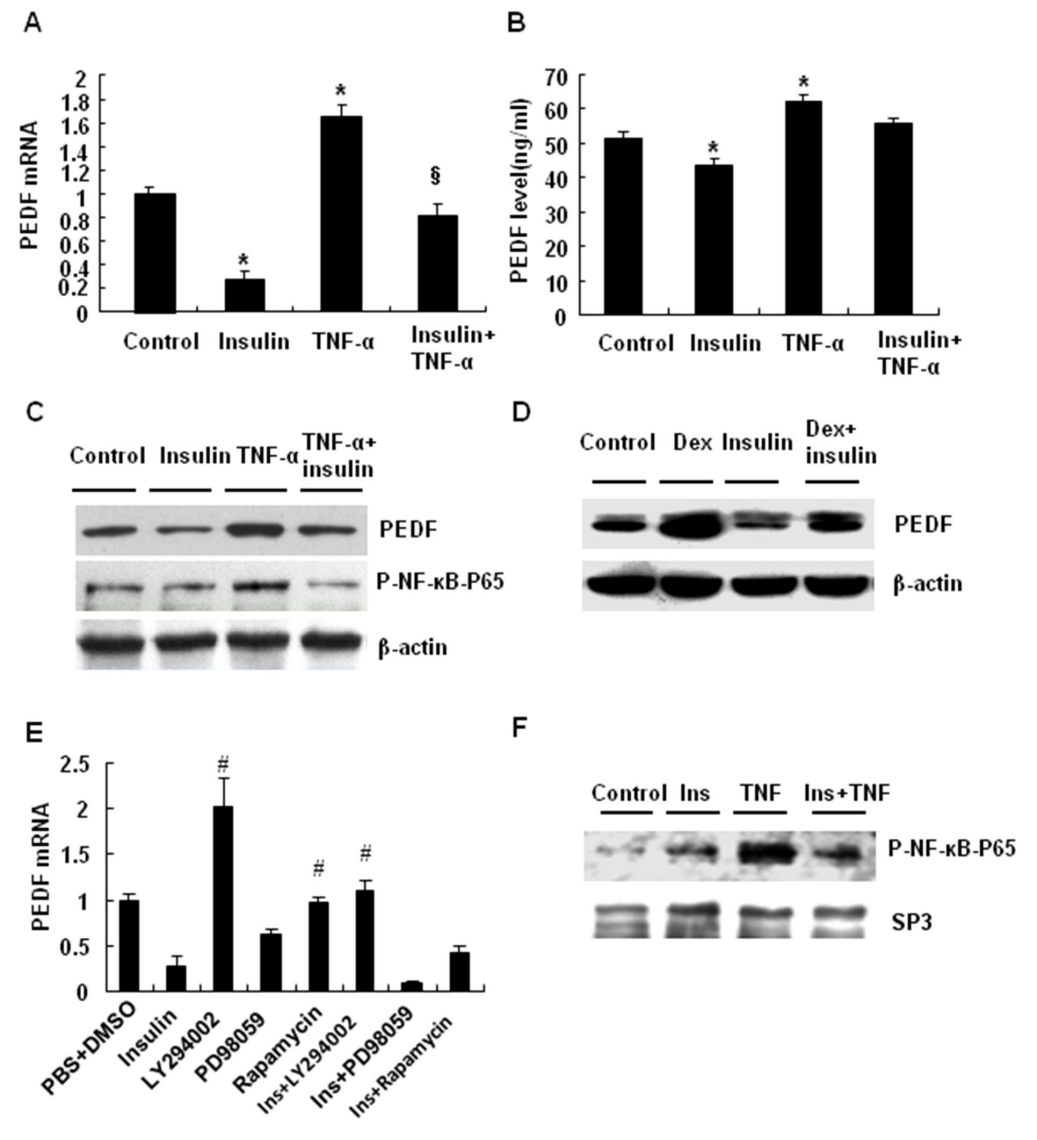
PEDF expression was inhibited by insulin in adipocytes. *A*: Insulin treatment decreased PEDF mRNA expression in 3T3-L1 cells. *B*: Insulin reduced PEDF protein and blocked the PEDF increase in response to TNF-α in 3T3-L1 cells. *C*: Insulin treatment decreased PEDF protein and reversed the TNF-α-induced PEDF protein in 3T3-L1 cells. *D*: PEDF expression in 3T3-L1 cells after treatment with Dex, insulin, or Dex+insulin. *E*: The signaling pathway for insulin-induced inhibition of PEDF mRNA expression in 3T3-L1 cells. The 3T3-L1 cells were treated with insulin, MEK inhibitor PD98059, PI3K inhibitor LY294002, or mTOR inhibitor rapamycin, alone or in combination for 24 h. “*”, “§” indicates P<0.05 compared with the control group and TNF-α group, respectively. “#” indicates P<0.05 compared with insulin treatment. *F*: The effect of insulin treatment on NF-κB expression in the nuclear extracts of 3T3-L1 cells. Insulin blocks the effects of TNF on NF-κB expression. The data are presented as the mean ± SEM of three to four independent experiments.

We investigated the molecular mechanism of PEDF expression in 3T3-L1 cells. 3T3-L1 cells were treated with TNF-α for 4 days or Dex for 8 days to induce insulin resistance. In those models, PEDF expression was induced by TNF-α and Dex. In response to TNF-α, the cells expressed more PEDF in mRNA and protein (Figure 3, A-C). Insulin reduced PEDF expression in the basal condition, and blocked the TNF-α effect (Figure 3, A-C). The PEDF expression was induced by Dex and the induction was suppressed by insulin (Figure 3D). These data suggest that PEDF expression is induced in adipocytes by TNF-α or Dex. Insulin is able to block PEDF expression in response to those treatments. 

We investigated the signaling pathway by which insulin inhibits PEDF expression in 3T3-L1 cells. Insulin activates two major signaling pathways: the phosphoinositide 3-kinase (PI3K) / protein kinase B (Akt) and methyl ethyl ketone (MEK) /ERK pathways. Inhibition of the PI3K/Akt pathway with LY294002, a selective PI3K inhibitor, blocked insulin activity in the inhibition of PEDF expression (P<0.05) (Figure 3E), indicating that the PI3K/Akt pathway is required for the insulin activity. LY294002 increased PEDF expression (P<0.05) in the absence of insulin, suggesting that the PI3K/Akt signaling pathway inhibits PEDF expression at the basal conditions. Inhibition of the MEK/ERK pathway with PD98059, a selective MEK inhibitor, did not affect the insulin activity (Figure 3E). Rapamycin, an inhibitor of the mammalian target of rapamycin (mTOR), had no effect on the insulin activity (P>0.1), suggesting that mTOR is not required for the insulin activity in 3T3-L1 cells (Figure 3E). NF-κB activity was investigated by examining p65 phosphorylation status. The phosphorylation was induced by TNF-α and the TNF-α effect was blocked by insulin (Figure 3F). 

### Induction of PEDF by TNF-α

**Figure 4:**
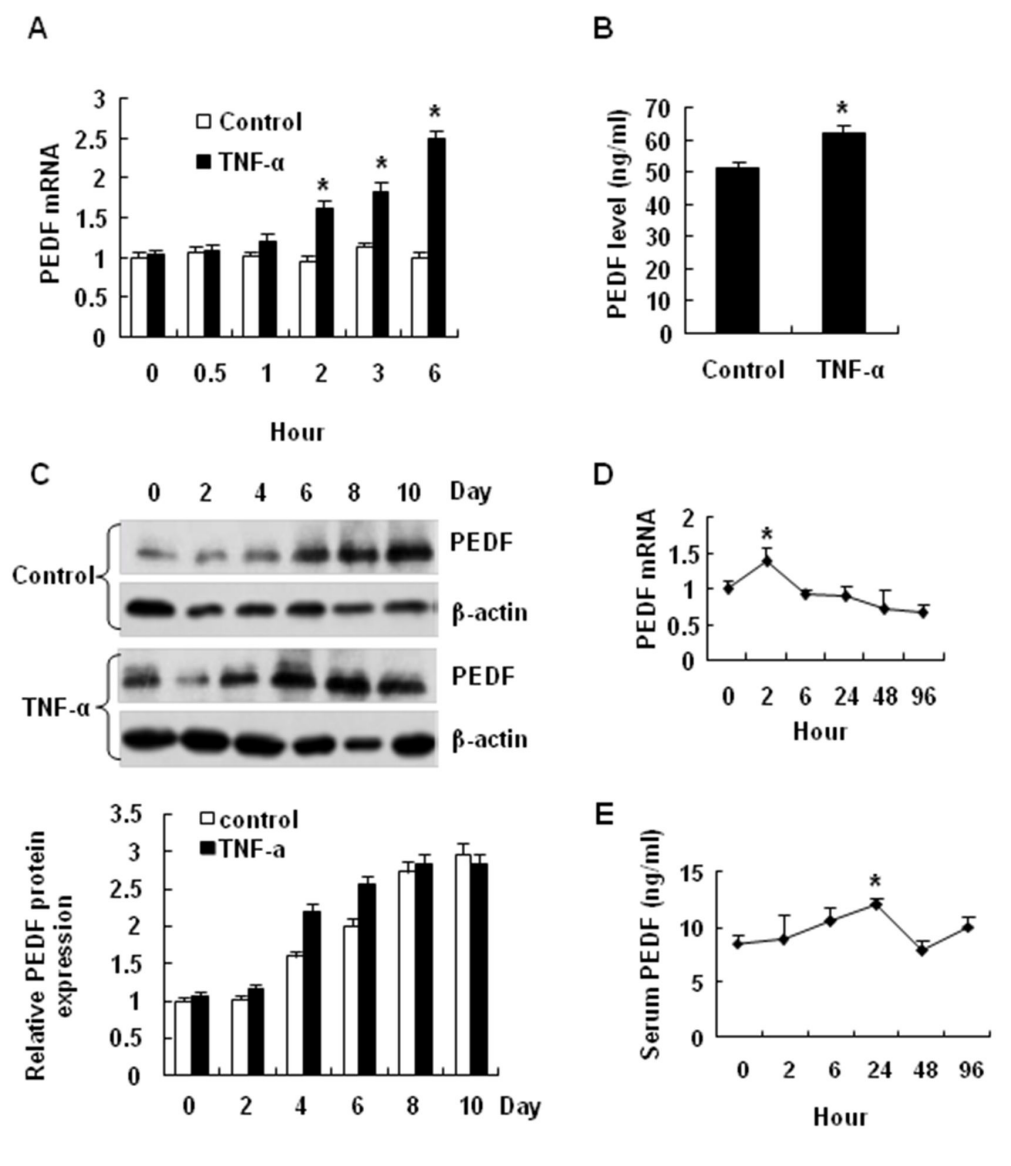
Induction of PEDF by TNF-α. *A*: PEDF mRNA expression in 3T3-L1 cells before and after TNF-α treatment at 30 min, 1, 2, 3, 6 hours in differentiated cells. *B*: PEDF levels in 3T3-L1 cell culture medium after TNF-α treatment for 48 hours. *C*: PEDF protein in differentiating 3T3-L1 cells with and without TNF-α treatment. Days of differentiation is indicated. *D*: The effects of TNF-α on PEDF mRNA expression in the adipocyte tissue of C57BL/6J mice. *E*: The effects of TNF-α on serum PEDF in C57BL/6J mice. The data are the mean ± SEM of four independent experiments. “*” indicates P<0.05 compared with the control group.

PEDF expression was further investigated in adipocytes in a time course study of TNF-α effect. In the un-differentiated cells, PEDF expression was increased by TNF-α in a time-dependent manner. The increase was observed in mRNA and protein (Figure 4, A and B). During differentiation of 3T3-L1, PEDF was increased at day 6 and the highest expression was observed at day 10 (Figure 4C). With TNF-treatment, the PEDF protein was increased earlier at day 4. The data suggests that PEDF expression is induced in 3T3-L1 adipocytes by TNF-α in both undifferentiated and differentiating conditions. 

We examined the TNF-α effect in vivo by determining PEDF expression in the epididymal fat tissue of C57BL/6J mice. With a single TNF-α injection, PEDF mRNA was transiently increased at 2 hours and then decreased thereafter in a 6 hour study (Figure 4D). The serum PEDF was significantly increased with a peak at 24 hours after the TNF-α injection (Figure 4E). The data suggest that PEDF expression is induced by TNF-α in vivo. 

### NF-κB Induces PEDF expression

**Figure 5:**
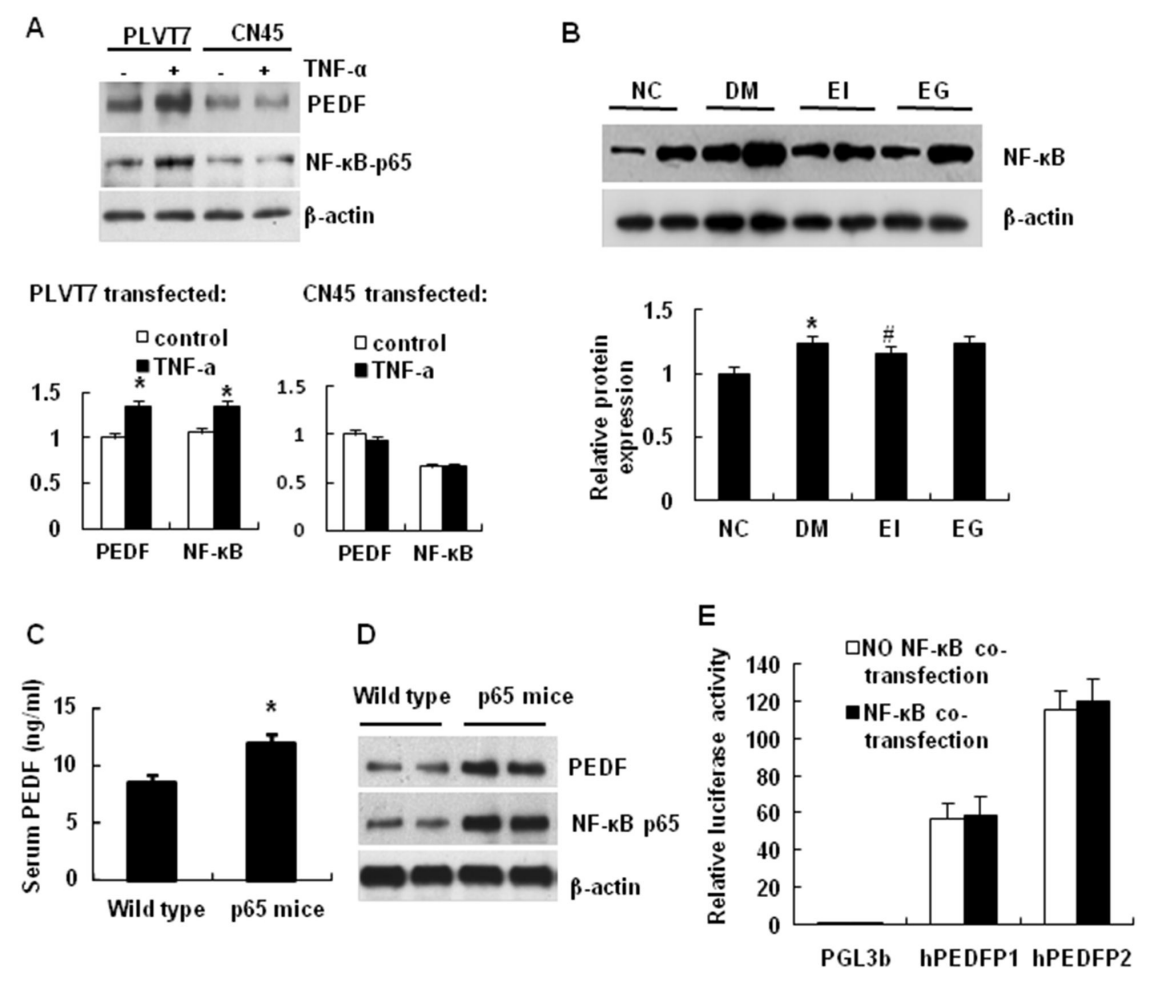
NF-κB induces PEDF expression. *A*: The effects of TNF-α on PEDF expression in 3T3-L1 cells after NF-кB-p65 knockdown. The lentivirus vector CN45 that expresses siRNA for NF-κB p65 was transfected into 3T3-L1 cells with Fugene HD. The control lentivirus vector is PLVT7. *B*: NF-κB p65 expression in the nucleus of adipose tissue of STZ-induced type 2 diabetic rats. *C*: Serum PEDF levels in wild type and aP2-p65 overexpressing mice. *D*: PEDF expression in aP2-p65 overexpressing mice. PEDF was determined in the epididymal fat tissue of aP2-p65 transgenic mouse by Weston blot. *E*: Effects of NF-κB on the PEDF promoter activities. hPEDFP1 (promoter does not respond directly to NF-κB activation. The data are the mean ± SEM of three to four independent experiments. “*” indicates P<0.05 compared with the control group. “#” indicates P<0.05 compared with diabetic group.

We investigated the signaling pathway by which TNF-α induces PEDF expression with a focus on NF-κB. We knocked down NF-κB p65 in 3T3-L1 cells using a vector-based interference RNA, which was delivered by lentivirus (CN45) into cells. The p65 knockdown decreased TNF-effect in the induction of PEDF expression (Figure 5A). In the control, the empty virus (PLVT7) had no effect. These data suggest that NF-κB activity is required for PEDF expression in the TNF-treated cells. The NF-κB activity was enhanced in the adipose tissue of diabetic rats and the NF-κB activity was reduced by the insulin therapy (Figure 5B). To test the role of NF-κB, we examined PEDF expression in the serum and adipose tissue of aP2-p65 mice, in which serum PEDF levels in ap2-p65 mice are significantly increased compared with the wild type mice, and NF-κB activity was enhanced in adipose tissue by overexpression of p65 subunit under the aP2 gene promoter. PEDF was significantly elevated in the transgenic mice relative to that of the wild type littermates (Figure 5, C and D). These data suggest that NF-κB induces PEDF expression, and TNF-α induces PEDF expression through NF-κB. The PEDF gene promoter was tested for a direct response to NF-κB in a reporter assay. Two promoter fragments (hPEDFP1, −1721/+38 and hPEDFP2, −4687/+38) were tested and their activities were not influenced by NF-κB in the co-transfection assay (Figure 5E). The data suggests that NF-κB may regulate PEDF expression through an indirect mechanism. 

### Regulation of 11β-HSD1 by NF-κB

**Figure 6:**
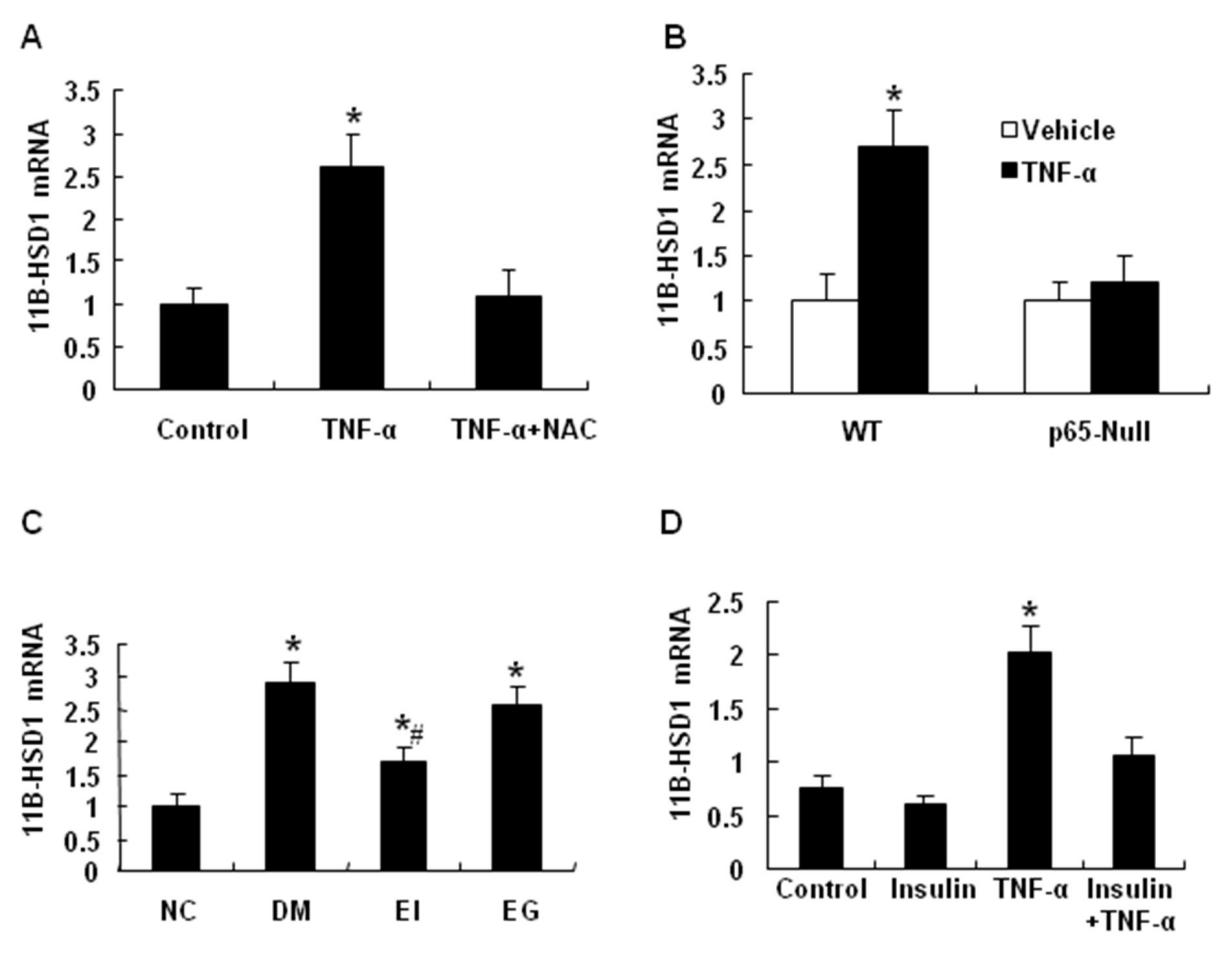
Regulation of 11β-HSD1 by NF-κB. *A*: The effects of the NF-κB inhibitor NAC on 11β-HSD1 expression in 3T3-L1 adipocytes. *B*: The effects of TNF-α on 11β-HSD1 mRNA expression in wild type MEF cells and the p65-null MEF cells. *C*: Insulin treatment decreased 11β-HSD1 mRNAs in the adipose tissue of type 2 diabetic SD rats. *D*: Insulin treatment blocked the increase in 11β-HSD1 mRNA in response to TNF-α treatment. The data are shown as the mean ± SEM of three independent experiments. “*” indicates P < 0.05 compared with the nondiabetic control group. “#” indicates P<0.05 compared with DM group. *NC*, normal control; *DM*, diabetic rats with no insulin therapy; *EI*, diabetic rats treated with insulin during early intervention study; EG, diabetic rats treated with gliclazide during early intervention study.

To understand the mechanism of NF-κB action, we tested 11β-HSD1 (11β-hydroxysteroid dehydrogenase type 1). 11β-HSD1 is an enzyme that generates the active form of glucocorticoids in cells. An increase in 11β-HSD1 activity contributes to the pathogenesis of insulin resistance, which has been demonstrated in mice by overexpression and ablation of 11β-HSD1 [21–23]. We observed that 11β-HSD1 expression was induced by TNF-α in 3T3-L1 adipocytes (Figure 6A). The induction was blocked by NF-κB inhibitor (Figure 6A), suggesting that 11β-HSD1 may be a target gene of NF-κB. To test this possibility, we examined 11β-HSD1 expression in p65-null MEF cells. TNF-α stimulated 11β-HSD1 expression in the wild-type MEF cells, but not in the p65-null MEF cells (Figure 6B). These data support that NF-κB is required for TNF-α induction of 11β-HSD1 expression. In the adipose tissue of diabetic rats, 11β-HSD1 expression was significantly increased in the diabetic group (DM) (P<0.01), and the expression was decreased by insulin treatment (EI) (P<0.05) (Figure 6C). Gliclazide had no significant effects on 11β-HSD1 expression in those animal models. Insulin treatment blocked the increase in 11β-HSD1 mRNA in response to TNF-α treatment (P<0.05) (Figure 6D). The data suggest that 11β-HSD1 is a target gene of NF-κB and its expression in the adipose tissue is inhibited by insulin treatment.

### PEDF induction by Dex

**Figure 7:**
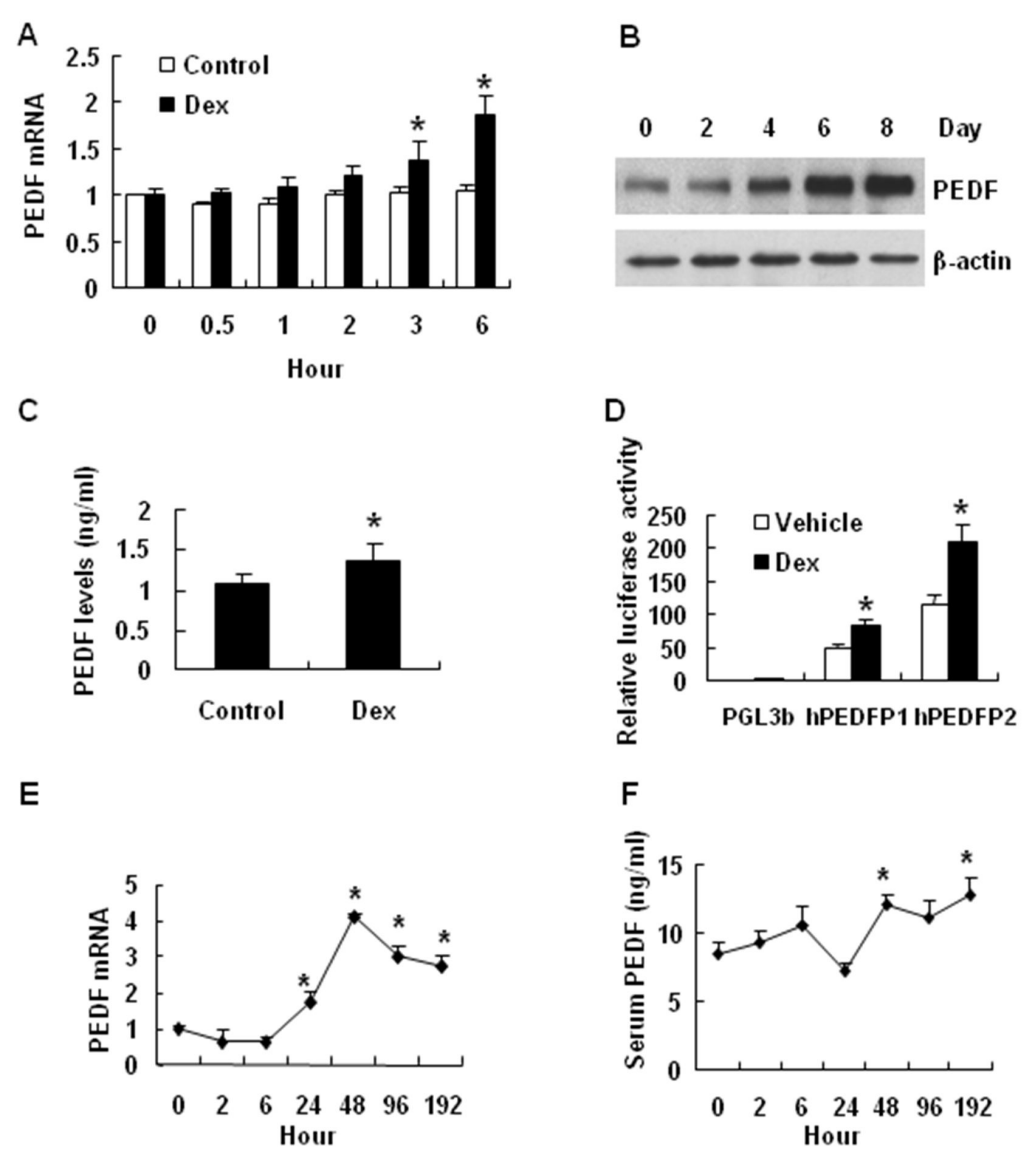
PEDF induction by Dex. The effect of Dex on the expression of PEDF was investigated in 3T3-L1 cells and C57BL/6J mice. *A*: The effects of Dex on PEDF mRNA expression in fully differentiated 3T3-L1 cells at 30 min, 1, 2, 3, 6 hours. *B*: PEDF expression in 3T3-L1 cells (fully differentiated) after Dex treatment at 2, 4, 6, and 8 days. *C*: The PEDF levels in the cell culture medium of 3T3-L1 cells after Dex treatment for 48 h. *D*: The effects of Dex on the PEDF promoter (hPEDFP1 and hPEDFP2) in 3T3-L1 cells. *E*: The effects of Dex on PEDF mRNA expression in the adipose tissue of C57BL/6J mice. *F*: The effects of Dex on serum PEDF in C57BL/6J mice. The data are shown as the mean ± SEM (n=4). “*” indicates P < 0.05 compared with control.

Dex is a synthetic and active form of glucocorticoid. Dex may contribute to PEDF expression during adipocyte differentiation as Dex is a component in the adipogenic cocktail. To test this possibility, we examined PEDF expression in cells treated with Dex (20 nM). The mRNA level was significantly increased by Dex at 3 hours in the treatment (P<0.05) (Figure 7A). Expression of PEDF protein was also increased as indicated by Western blot data from the whole cell lysate and ELISA data from the cell culture medium (P<0.05) (Figure 7, B and C). These data suggest that Dex induces PEDF expression at mRNA and protein levels. The molecular mechanism was investigated using the PEDF luciferase reporter in a transient transfection assay. The activity of long promoter (hPEDFP2, -4687 bp) was increased by 81.0% in 3T3-L1 cells treated with Dex, while the activity of short promoter (hPEDFP1, -1721 bp) was increased by 74.5% (Figure 3F). These data suggest that both promoters contain a Dex-responsive fragment (Figure 7D). This activity of Dex was tested in mice by injection of Dex. PEDF mRNA expression was induced in the adipose tissue of Dex-treated mice (Figure 7E). The PEDF protein was elevated in the serum of the same mice (Figure 7F) (P<0.01). The data suggest that PEDF expression is induced by glucocorticoid both in vitro and in vivo. 

## Discussion

In this study, we investigated the mechanism of insulin sensitization of insulin therapy by examining the anti-angiogenic factor PEDF in type 2 diabetic patients. We observed that insulin therapy decreased blood PEDF in type 2 diabetic patients. PEDF that was originally identified in the human retinal pigment epithelial cells [2] is the most potent angiogenic inhibitor in mammalian eye [7,24]. PEDF expressed in adipocytes is involved in the pathogenesis of insulin resistance. Blood PEDF is increased in type 2 diabetic patients [11,12,14,25,26], and is positively associated with the degree of insulin resistance [27]. Blood PEDF is decreased by weight loss [28], which is known to enhance insulin sensitivity. Genetic study suggests that variant in the coding region of PEDF gene (SNP rs12603825) is associated with insulin resistance in obesity [29]．The mechanism by which PEDF contributes to insulin resistance remains largely unknown. Inhibition of angiogenesis in adipose tissue is likely a mechanism that explains PEDF activities in blocking adipogenesis [10,15], stimulating lipolysis [9] and promoting inflammation [30].  Vascular endothelial growth factor (VEGF) is an important regulator of angiogenesis. There is increased ratio of VEGF/PEDF in ischemia-induced retinal neovascularization in rats [31]. One of the possible mechanisms by which PEDF could down-regulate angiogenesis is that PEDF inhibit migration and tube formation of endothelial cells by abrogating VEGF-mediated activation of PI3K/Akt [32]. However, there is no report about PEDF response to insulin therapy in the diabetic patients. In this study, we addressed this issue by testing PEDF alteration in the patients after insulin therapy. Blood PEDF was reduced by insulin in the diabetic patients (Figure 1) and the result was confirmed in diabetic rats (Figure 2). PEDF expression was decreased in the adipose tissue of rats after insulin therapy (Figure 2B). In 3T3-L1 adipocyte, insulin suppressed PEDF expression (Figure 3). The data suggests that the beneficial effect of insulin is to reduce PEDF availability in circulation by inhibiting PEDF expression in adipose tissue.

In this study, we compared insulin and gliclazide in the regulation of PEDF in diabetic rats. We found that gliclazide did not change PEDF although gliclazide induces insulin secretion in the control of blood glucose. The difference may be due to protection of β-cell function by insulin treatment, which leads to a significant increase in blood insulin in response to glucose challenge (Table S1). Gliclazide does not show this effect although it could induce insulin secretion. Under T2D condition, β-cells produce more insulin to compensate for insulin resistance. This response causes β-cells overstimulation and dysfunction. Intensive insulin therapy of newly diagnosed T2D could decrease the secretory demand and protect β-cell function, while sulfonylurea such as gliclazide may accelerate β-cell dysfunction through inducing insulin secretion and over stimulating β-cells.

The current study provides a new mechanism for PEDF elevation in obesity. PEDF is expressed in adipocytes and adipose tissue. The expression is increased in obesity and diabetic conditions. However, it is largely unknown what induces PEDF expression in those conditions. We addressed this issue by studying PEDF expression in 3T3-L1 adipocytes. We observed that PEDF expression was induced by TNF-α, and the response was depended on NF-κB activation. Knockdown of NF-κB p65 decreased PEDF expression in adipocytes and over expression of NF-кB p65 enhanced PEDF expression in the adipose tissue of aP2-p65 mice (Figure 5). However, our data suggests that NF-кB does not directly regulate the PEDF gene promoter in a cotransfection study (Figure 5D). Instead, NF-кB induced 11β-HSD1 expression, which promotes synthesis of bioactive glucocorticoids. These intermediate events are likely responsible for the disassociation of NF-κB activity and PEDF expression in adipose tissue (Figures 2 and 5).

PEDF may mediate the glucocorticoid activity in the pathogenesis of insulin resistance. Glucocorticoids have strong activities for insulin resistance, anti-inflammation and anti-angiogenesis. We observed that dexamethasone induced PEDF expression in adipose tissue, and in mechanism, dexamethasone activated PEDF gene promoter activity in adipocytes (Figure 7). The observations suggest that PEDF may mediate the insulin resistant activity of glucocorticoid. Glucocorticoid induces gene transcription through activation of glucocorticoid receptor in the nucleus. This mechanism may account for activation of PEDF gene promoter and gene expression in adipocytes in obesity. This possibility remains to be tested by identifying the glucocorticoid response element in the PEDF gene promoter. The connection of glucocorticoid and PEDF provides a new mechanism for glucocorticoid activities in insulin resistance. 

Our data suggests that insulin may inhibit PEDF expression in adipocytes by targeting 11β-HSD1/glucocorticoid pathway. In this study, we observed that insulin decreased 11β-HSD1 expression although the mechanism remains unknown. 11β-HSD1 expression is induced by NF-кB and decreased by HIF-1α [33]. Insulin may downregulate 11β-HSD1 by inhibiting NF-κB activation in cells treated with TNF-α (Figure 3). Alternatively, insulin may suppress 11β-HSD1 expression by activation of HIF-1. Insulin induces HIF-1 activity through activation of PI3K/Akt pathway [34]. Inhibition of 11β-HSD1 by insulin may contribute to insulin sensitization through a reduction in glucocorticoid, which is a risk factor for insulin resistance. Glucocorticoid impairs insulin sensitivity through multiple pathways [35]. Inhibition of 11β-HSD1/glucocorticoid pathway represents a new mechanism by which insulin improves insulin sensitivity. 11β-HSD1 contributes to insulin resistance as suggested by gene knockout and over expression studies in mice [21,22]. The current study suggests that PEDF may mediate 11β-HSD1 activity in the pathogenesis of insulin resistance. It is also possible that insulin directly regulates PEDF expression by blocking glucocorticoid activity. It is worth to explore the possibility in the future. 

In summary, we observed that insulin reduced blood PEDF in type 2 diabetic patients. In vivo, insulin inhibits PEDF expression in the adipose tissue of diabetic rats. In vitro, insulin suppresses PEDF expression in adipocytes. PEDF expression is induced by inflammation through an indirect mechanism that may involve 11β-HSD1 expression from NF-κB activation. 11β-HSD1 may promote production of active glucocorticoid in the induction of PEDF. PEDF is likely to mediate glucocorticoid activity in the pathogenesis of insulin resistance. Insulin inhibits expression of two insulin resistance-related genes, PEDF and 11β-HSD1. This insulin activity represents a novel mechanism of insulin sensitization in the insulin therapy. In addition, we provide evidence about regulation of PEDF by TNF-α and glucocorticoid although the detail molecular events remain to be identified. 

## Supporting Information

Figure S1 **Body weight and glucose levels of the Sprague-Dawley rats.** ^a^ P<0.05 and ^b^ P<0.01 compared with values for normal control rats. ^c^ P<0.05 and ^d^ P<0.01 compared with values for diabetic rats with no therapy. *NC* normal control, *EDM* diabetic rats with no therapy, *EI* diabetic rats treated with insulin, *EG* diabetic rats treated with gliclazide. (Previously published data in Acta Diabetol (2008) 45:167–178). .
(PPTX)

Table S1 **Characteristics of the Sprague-Dawley rats without and with insulin treatment.** NC normal control, DM diabetic rats with no therapy, EI diabetic rats treated with insulin during early intervention study, EG diabetic rats treated with gliclazide during early intervention study. Compared with NC group, ^a^ P< 0.05 and ^b^ P< 0.01; Compared with DM group, ^c^ P< 0.05 and ^d^ P< 0.01. (Previously published data in Acta Diabetol (2008) 45:167–178). .
(PPTX)
