# Supplementary material for: PEDF Expression Is Inhibited by Insulin Treatment in Adipose Tissue via Suppressing 11β-HSD1
Source: PLoS One. 2013 Dec 18;8(12):e84016. doi: 10.1371/journal.pone.0084016 (PMC3867502; doi:10.1371/journal.pone.0084016)
Supplement: Figure S1 — Body weight and glucose levels of the Sprague-Dawley rats. a P<0.05 and b P<0.01 compared with values for normal control rats. c P<0.05 and d P<0.01 compared with values for diabetic rats with no therapy. NC normal control, EDM diabetic rats with no therapy, EI diabetic rats treated with insulin, EG diabetic rats treated with gliclazide. (Previously published data in Acta Diabetol (2008) 45:167–178). . (PPTX) [file pone.0084016.s001.pptx]

## Slide 1
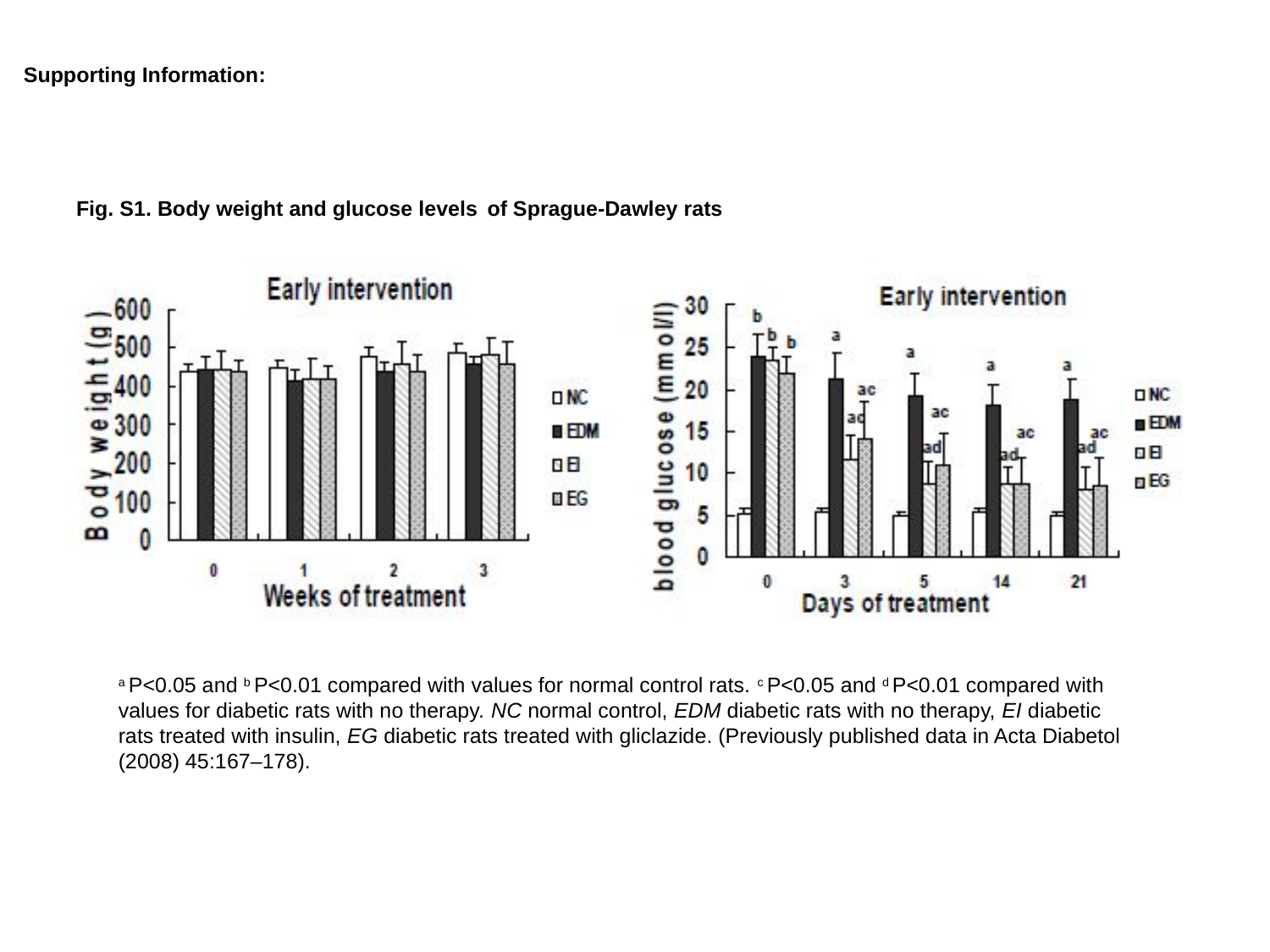

Supporting Information:
Fig. S1. Body weight and glucose levels of Sprague-Dawley rats
a P<0.05 and b P<0.01 compared with values for normal control rats. c P<0.05 and d P<0.01 compared with values for diabetic rats with no therapy. NC normal control, EDM diabetic rats with no therapy, EI diabetic rats treated with insulin, EG diabetic rats treated with gliclazide. (Previously published data in Acta Diabetol (2008) 45:167–178).
