# Supplementary material for: PEDF Expression Is Inhibited by Insulin Treatment in Adipose Tissue via Suppressing 11β-HSD1
Source: PLoS One. 2013 Dec 18;8(12):e84016. doi: 10.1371/journal.pone.0084016 (PMC3867502; doi:10.1371/journal.pone.0084016)
Supplement: Table S1 — Characteristics of the Sprague-Dawley rats without and with insulin treatment. NC normal control, DM diabetic rats with no therapy, EI diabetic rats treated with insulin during early intervention study, EG diabetic rats treated with gliclazide during early intervention study. Compared with NC group, a P< 0.05 and b P< 0.01; Compared with DM group, c P< 0.05 and d P< 0.01. (Previously published data in Acta Diabetol (2008) 45:167–178). . (PPTX) [file pone.0084016.s002.pptx]

## Slide 1
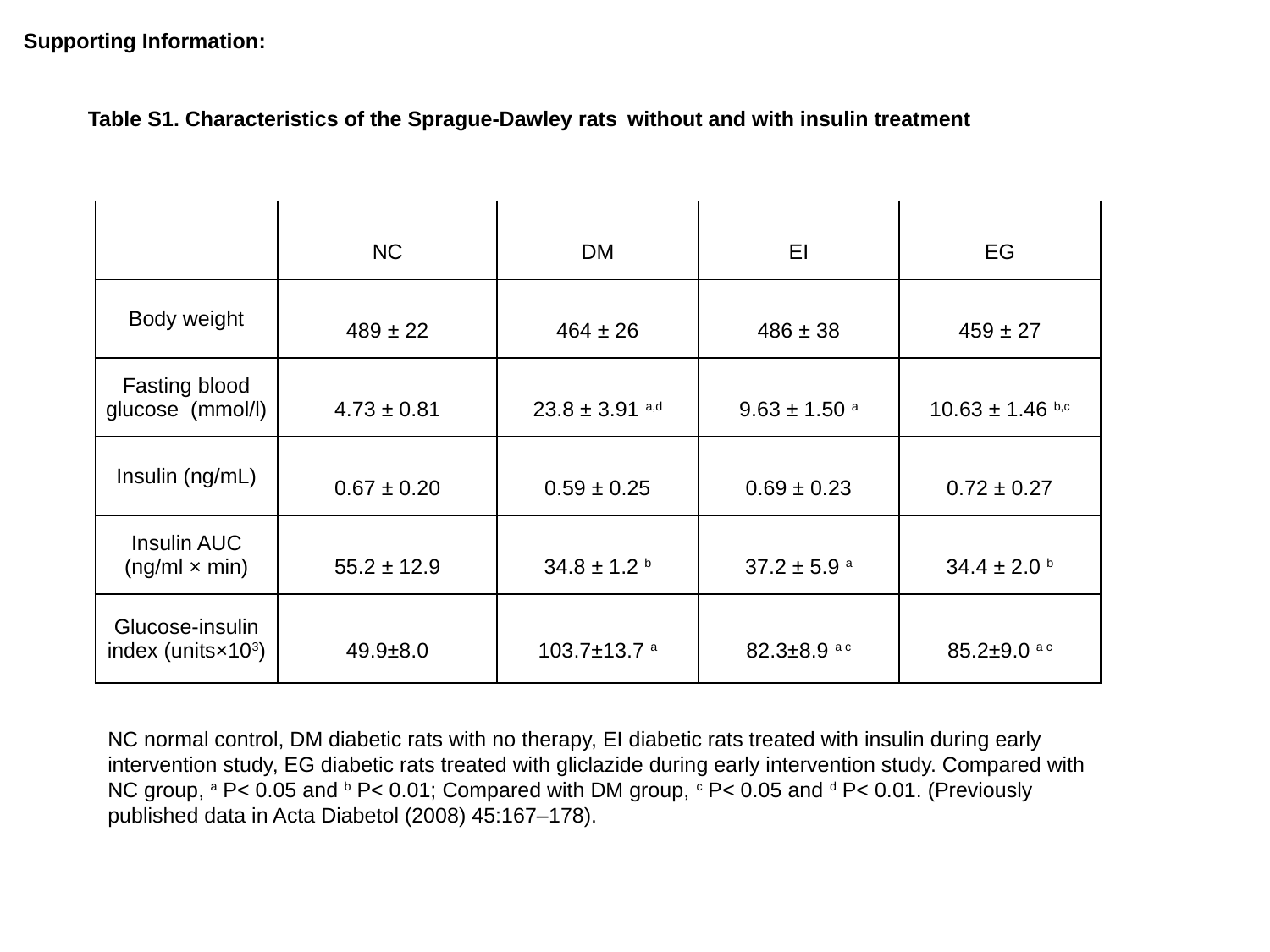

Supporting Information:
Table S1. Characteristics of the Sprague-Dawley rats without and with insulin treatment
| | NC | DM | EI | EG |
| --- | --- | --- | --- | --- |
| Body weight | 489 ± 22 | 464 ± 26 | 486 ± 38 | 459 ± 27 |
| Fasting blood glucose (mmol/l) | 4.73 ± 0.81 | 23.8 ± 3.91 a,d | 9.63 ± 1.50 a | 10.63 ± 1.46 b,c |
| Insulin (ng/mL) | 0.67 ± 0.20 | 0.59 ± 0.25 | 0.69 ± 0.23 | 0.72 ± 0.27 |
| Insulin AUC (ng/ml × min) | 55.2 ± 12.9 | 34.8 ± 1.2 b | 37.2 ± 5.9 a | 34.4 ± 2.0 b |
| Glucose-insulin index (units×103) | 49.9±8.0 | 103.7±13.7 a | 82.3±8.9 a c | 85.2±9.0 a c |
NC normal control, DM diabetic rats with no therapy, EI diabetic rats treated with insulin during early intervention study, EG diabetic rats treated with gliclazide during early intervention study. Compared with NC group, a P< 0.05 and b P< 0.01; Compared with DM group, c P< 0.05 and d P< 0.01. (Previously published data in Acta Diabetol (2008) 45:167–178).
